# How clinicians make (or avoid) moral judgments of patients: implications of the evidence for relationships and research

**DOI:** 10.1186/1747-5341-5-11

**Published:** 2010-07-09

**Authors:** Terry E Hill

**Affiliations:** 1 Department of Medicine, University of California, San Francisco, USA

## Abstract

Physicians, nurses, and other clinicians readily acknowledge being troubled by encounters with patients who trigger moral judgments. For decades social scientists have noted that moral judgment of patients is pervasive, occurring not only in egregious and criminal cases but also in everyday situations in which appraisals of patients' social worth and culpability are routine. There is scant literature, however, on the actual prevalence and dynamics of moral judgment in healthcare. The indirect evidence available suggests that moral appraisals function via a complex calculus that reflects variation in patient characteristics, clinician characteristics, task, and organizational factors. The full impact of moral judgment on healthcare relationships, patient outcomes, and clinicians' own well-being is yet unknown. The paucity of attention to moral judgment, despite its significance for patient-centered care, communication, empathy, professionalism, healthcare education, stereotyping, and outcome disparities, represents a blind spot that merits explanation and repair. New methodologies in social psychology and neuroscience have yielded models for how moral judgment operates in healthcare and how research in this area should proceed. Clinicians, educators, and researchers would do well to recognize both the legitimate and illegitimate moral appraisals that are apt to occur in healthcare settings.

## Introduction

In 1926 Francis Peabody ended his most celebrated lecture with the oft-repeated conclusion, "the secret of caring for the patient is caring for the patient" [1]. It's a compelling line, resonant with wisdom and common sense, but it begs an obvious question. What if I don't care for the patient? In particular, what if my reaction to the patient is negative, perhaps intensely so, driven by social and/or moral disapproval? This last question arises occasionally in bioethics and "difficult patient" discussions, but beyond assertions as to what *should *happen, there is little systematic data on what *actually *happens.

Most healthcare professionals have found themselves treating someone who is flagrantly offensive, whose attitudes and actions have caused others to suffer harm. Physicians and nurses readily admit that empathy is more difficult to achieve in these situations and that their professional ideals feel strained. Two published reports will illustrate:

Renate Justin's new patient with emphysema revealed during her intake history that she was an unrepentant Nazi anti-Semite who had supervised slave laborers during the war. Justin, a Jewish family physician, struggled through the turmoil of her feelings and duties before the second visit.

 "I had decided that if she stayed with my practice, I could probably be a skilled and trustworthy physician to her. Intellectually, I had concluded that my job as a doctor was to take care of her, regardless of her history. I felt that I could achieve this: I could treat her emphysema and suppress or control my moral outrage. What I did not know was whether I could be compassionate." [2]

In a 2004 account of a Midwestern surgical intensive care unit, anthropologist Joan Cassell found that physicians, male and female, tried to avoid thinking about their patients' personal stories. Not so the nurses.

"The nurses always know the patients' stories: the accidents, tragedies, and sorrows that brought them to the hospital, their family constellations, and their clashes with others and, on occasion, with the criminal justice system. As a result, however, some nurses make harsh moral judgments."

They nurses freely disparaged criminals, as well as the woman who was thought to be loaded on heroin and alcohol when she failed to fasten a seatbelt on her three-year-old son, with devastating results [3].

In the first example, Dr. Justin girded herself so as to prevent negative consequences to her patient, a cognitive and emotional maneuver that comes with a cost to clinician wellbeing. In the surgical ICU, as Cassell documents, it was the patients who reaped the most obvious negative consequences of clinicians' judgments.

The prominence of moral judgment in such egregious situations is self-evident, but this review will stake out the broader claim that moral emotions and judgments are active in everyday clinical encounters. The role of moral judgment is largely unrecognized in the literatures on healthcare communication, caring, empathy, trust, disparities, and education. Yet since the mid-twentieth century, sociologists have noted the prevalence of moral judgment in healthcare. And increasingly over the last decade, social psychologists and neuroscientists have produced a rich body of work on moral emotions and cognitions that promises to reframe our understandings of morally charged clinical relationships. This review will survey these literatures and the scant empirical data from physician and nurse researchers that are relevant to moral judgment, highlighting the variation that emerges from diverse combinations of patients, clinicians, tasks, and settings, as well as the most promising research strategies.

## Methods

The search for studies on moral judgment in healthcare was largely disappointing, even within the literatures on empathy and caring. The literature on difficult clinical relationships is extensive, but moral judgment is at best a secondary focus in these studies. Research on attitudes to stigmatized conditions such as obesity, substance abuse, and self-harm is limited but relevant. Using a variety of PubMed search strategies, *e.g*., with MESH terms "morals," "empathy," "physician-patient relations," and "nurse-patient relations," I retrieved 23,240 English-language references. Of these, I identified 400 potentially useful articles. Manual searches of these led to additional articles and searches in snowball fashion. Of the 2000 articles I eventually examined, less than a hundred offered any pertinent data from healthcare settings.

Given the paucity of research on moral dynamics in healthcare relationships, this review is an exploratory exercise in "sensemaking," which Karl Weick likens to cartography [4]. I worked with my final collection of articles like a dataset, sorting and coding the observations and results in constant comparative fashion, testing my categories against the data, my experience, and my own interviews, seeking to identify at least the major landmarks in this neglected terrain. Tables 1 and 2 illustrate the breadth and creativity of methodologies employed in the reviewed studies from healthcare and social psychology, respectively.

**Table 1:** Methodologies of the healthcare studies discussed.

Ethnography, participant observation, qualitative interviews, focus groups	Cassell, 2004 [3]; Zola, 1963 [8]; Glaser, 1964 [9]; Roth, 1972 [10]; Varcoe, 2003 [12]; Emerson, 1976 [16]; Jeffery, 1979 [18]; Bolton, 2005 [19]; Willems, 2005 [40]; Monnickendam, 2007 [45]; Fiscella, 1997 [46]; May, 2004 [48]; Wear, 2006 [50]; Hadfield, 2009 [54]; Lyth, 1988 [60]
Survey of clinicians or patients (not both)	Weitzman, 2000 [44]; Malat, 2006 [47]; Mackay, 2005 [53]; Nicolaidis, 2005 [56]; Regan, 2009 [61]; Foster, 2003 [64]; von Hippel, 2008 [66]; Merrill, 1993 [87]

Mixed survey and qualitative interviews	Bowers, 2002 [20]; Regehr, 2002 [129]

Linked qualitative physician and patient interviews	Scott, 2008 [26]

Linked patient and physician surveys with one-year follow-up patient survey	Hall, 2002 [13]

Videotaped visits followed by linked qualitative interviews of patients and clinicians	Katz, 2009 [14]

Observed and audiotaped visits followed by linked qualitative interviews of patients and clinicians	Weissmann, 2006 [110]

Qualitative physician interviews linked to patient record review	Shaw, 2004 [17]

Survey of physicians linked to patient records and angiogram data	van Ryn, 2006 [71]

Standardized patient visits (surreptitiously audiotaped) linked to psychological testing of physicians	Chapman, 2008 [88]

Psychological tests and speech analysis of patients linked to psychological tests and speech analysis of clinicians	Berry, 2008 [93]

Tests of implicit and explicit attitudes linking clinicians and patients (IAT)	Brener, 2007 [65]

Conversation analysis of videotaped visits	Webb, 2009 [51]; Pillet-Shore, 2006 [52]

Web-based trigger written vignette and photo followed by survey and implicit attitude tests (IAT)	Green, 2007 [70]

Trigger videotape followed by countertransference instrument	Schwartz, 2007 [59]

Educator interviews of trainees following observed visits	Smith, 2005 [62]

Trigger written vignette followed by survey and empathy instrument	Tait, 2005 [49]

Controlled experiment randomizing medical students into different teaching programs with quantitative and qualitative performance, psychological, and sociological data	Hammond, 1959 [42]

The first cluster encompasses a diverse group of qualitative methodologies, often mixed within the same study. The survey methodologies in the second cluster also vary significantly. Of note are studies that link specific clinicians to data on their specific patients. IAT = Implicit Association Test.

**Table 2:** Methodologies of illustrative social psychology and neuroscience studies discussed.

Web-based survey (psychological instruments including vignette trigger) of students and managers	Reynolds, 2007 [28]
Functional MRI responses and lexical priming tests of stereotype activation to trigger photographs (black and white faces) under three different social task conditions	Wheeler, 2005 [30]

Functional MRI and emotional responses to trigger photographs illustrating different social groups and objects	Harris, 2006 [31]

Web-based international survey using measures of beliefs, attitudes, and stereotypes	Oldmeadow, 2007 [32]

Moral judgment responses to vignettes under four different conditions stimulating disgust and neutral controls	Schnall, 2008a [33]

Functional MRI responses to moral and non-moral stimuli of disgust	Borg, 2008 [34]

Moral judgment responses to heterosexual and homosexual couples and completion of disgust sensitivity scale; IAT response to heterosexual and homosexual couples	Inbar, 2009a [35]

Web-based surveys of disgust sensitivity, political orientation and political attitudes	Inbar, 2009b [36]

Moral judgment responses to vignettes following verbal priming for cleanliness or control and following hand-washing or control	Schnall, 2008b [37]

Measures of automatic evaluations of a person given various positive and negative information, photos with changing backgrounds, and affective priming	Rydell, 2009 [73]

Measure of egalitarian commitment and priming test of preconscious stereotype activation	Moskowitz, 1999 [75]

Functional MRI responses to written scripts stimulating specific moral emotions	Moll, 2007 [79]

Measures of subjective autonomic awareness, skin conductance, heart rate, and behavior of subjects in an immersive virtual environment who were told to give electric shocks to a virtual (not real) stranger	Slater, 2006 [84]

Functional MRI responses while playing video games	King, 2006 [85]

Measures of emotional response, generosity, and oxytocin (and 4 other hormones) in response to emotional videos	Barraza, 2009 [98]

Functional MRI responses to unpleasant pictures, including moral violations	Harenski, 2008 [106]

Measures of expressive behavior, subjective feelings, and physiology (pulse, finger temperature, skin conductance, heart rate and somatic activity) in response to disgusting film stimuli under different instructions to control *versus *suppress feelings	Gross, 1998 [121]

This table highlights the diversity of research methodologies. MRI = magnetic resonance imaging, IAT = Implicit Association Test.

### The organizational and reciprocal dynamics of difficult relationships

The difficult patient is one who "makes me feel ineffective," according to a 1958 study that canvassed a clinic's physicians, nurses, social workers, and clerical workers [5]. Patients who fail to validate clinicians' sense of themselves as effective professionals, who threaten their control, and/or who create fruitless work are all subject to being labeled "bad patients." Kelly and May noted these themes of validation, compliance, and efficiency in a 1982 review, as well as the methodological weaknesses and "high moral tone" that still pervade this literature [6]. A review by John Eisenberg sounded similar themes, noting that "much of the literature in this field consists of normative descriptions of how a physician should behave. Further investigation is needed into how the clinician does behave" [7].

The best naturalistic studies of how clinicians *actually *behave come from sociologists. In a 1963 report, Irving Zola found that the treatment of medically unexplained symptoms in three clinics at Massachusetts General Hospital varied by patient ethnicity, physician specialty, the spatial layout of the clinic, and the path sequence of patient contact with physicians and ancillary personnel [8]. The physicians might have described these patients as "difficult," but Zola focused on the organizational factors of care that significantly influenced complex reciprocal interactions between patients and physicians, as well as the ethnic differences (Italian and Irish) in how these lower-class patients presented their symptoms.

The classic sociological studies showed no reticence in reflecting ascribed social and/or moral worth of patients. According to Glaser and Strauss, "In our society we value people, more or less, on the basis of various social characteristics: for example, age, skin color, ethnicity, education, occupation, family status, social class, beauty, 'personality,' talent, and accomplishments" [9]. Their study found that nurses judged dying patients by their perceived social loss, often giving "more than routine care" to higher status patients and "less than routine care" to the unworthy. "People dying from a Friday night knife fight, or the adolescent on the verge of death who has killed others in a wild car drive, have their own social loss reinforced by an 'it's their own fault' rationale."

Julius Roth's extended study of six emergency departments, published in 1972, found that hospital staff "make judgments about the worthiness of the person and the appropriateness of his demands and take these judgments into account when performing the service" [10]. Several of Roth's findings are pertinent. First, he emphasized that staff will take moral judgments into account "*unless discouraged from doing so by the organizational arrangements under which they work*" (italics in original). Also, "We observed hints that certain ethnic groups are discriminated against, but this is very difficult to detect nowadays because everyone is extremely sensitive to the possibility of accusations of racial discrimination." These observations suggest that organizational norms do have impacts on staff's moral judgments and/or behavior. Consistent with the clinician effectiveness theme cited above, in straightforward surgical cases "the characteristics and behavior of the patient can be largely ignored."

Roth detailed how patients acquire labels, sometimes even before arriving at the hospital, *e.g*., when police or paramedics describe someone, more or less accurately, as a "drunk." Labeling is not always straightforward or consistent, however, as more recent research demonstrates. An ethnographic study on a nursing unit found that patients could acquire a likable label even when they were non-compliant or demanding or had a stigmatizing illness [11]. Furthermore, their labels could vary from nurse to nurse and over time, and nurses could hold multiple appraisals of the same patient. Demographic data are not necessarily a patient's destiny. In a rich ethnographic meta-analysis from three nursing units, Colleen Varcoe and colleagues elaborated on the contextual dynamics of labeling [12]. Stereotypical thinking, moral judgments, and coercive behavior increased in the context of time pressures and limited resources, whether actual or perceived. Their analysis showed, however, that this justificatory "ideology of scarcity" derives not only from higher management and policy arenas, but also from the local organizational microculture, sustained by the nurses' own matrix of personal relationships.

Judith Hall and colleagues revealed the reciprocity of "liking" by surveying both patients (diabetics at Kaiser) and physicians immediately after a visit [13]. The patients and physicians were able to gauge whether the other liked them, and that perception predicted whether they themselves liked the other. Physicians liked their healthier patients more than their sick patients, and healthier patients liked their physicians more. Physician liking predicted patient satisfaction a year later. Katz and Alegria examined the reciprocity of appraisals in greater detail by analyzing videotapes of visits together with linked qualitative interviews of patients and clinicians in a mental health clinic, revealing the social and moral judgments involved [14]. This triangulation of data allowed the researchers to document precise moments in which clinicians dropped their stereotypes and became fully present with their patients. Their patients clearly recognized and responded to those moments of engagement.

### Dirty work expertise

The "dirty work" literature [15] further explores the contextual dynamics of moral judgment. Dirty work tends to be designated as such because it is inherently "odious and onerous" and often ineffective, as described in a study of community psychiatric emergency intervention [16]. This literature confirms and extends the earlier finding that patients who fail to legitimize clinicians' effectiveness acquire negative labels. Patients with mental illness, for instance, frequently represent dirty work to primary care physicians, whereas moral or social judgments may not matter when the same patients present with remediable problems [17]. As one physician gleefully noted, "I enjoy anything which involves bone-setting, plastering, stitching, draining pus" [18].

Clinicians doing dirty work may be able to transform their chosen fields, however tainted and stigmatized, into grounds for pride in their expertise and commitment. Sharon Bolton has described the extraordinary moral complexity of nurses on a gynecology unit devoted to failed pregnancies, abortions, cancer, incontinence, and sexually transmitted disease [19]. Experienced nurses in such settings may hold and express strongly ambivalent feelings and judgments toward patients as well as toward inexperienced nurses and the physician staff. In this study, the nurses described a process of developing both task and emotional expertise over time, *e.g*., in getting past their sometimes intense physical repugnance by focusing on details of the process at hand. The uniqueness of their challenges and expertise fostered group cohesion, which helped to sustain them individually and to diminish the salience of moral issues.

Len Bowers has studied individual and organizational factors associated with positive attitudes toward work in three institutions for criminals with dangerous and severe personality disorders [20-22]. In order to manage their anger, frustration, disgust, and repulsion, nurses used a variety of strategies, including high moral and professional commitments, deep psychosocial understandings of the patients, and appropriately limited expectations. They also relied on interpersonal, team, and organizational supports, leading Bowers to recommend sustained managerial commitment to supportive treatment philosophies, operational practices, training, and supervision.

### The limits and value of professionalism

Apart from recommendations as to how clinicians should behave, the literatures on bioethics and professionalism are largely silent as to the frequency, impact, and dynamics of moral appraisals. Mary Catherine Beach and colleagues have argued that physicians' moral obligation includes "*recognition of the unconditional value of patients as persons*" (italics in original) [23]. While conceding that feeling respect for all patients may require "internal work," they did not describe that internal work or address how it could be accomplished. Jones and McCullough, considering the case of a death row inmate with HIV and indications for abdominal aneurysm repair, simply asserted that the surgeon should proceed with the repair because "ethical obligations to all patients needing your care do not vary with their character, social histories, belief systems, or other features unrelated to their medical condition" [24].

Such exhortations gloss over the internal and interpersonal challenges described, for instance, in Groves' landmark article about "hateful patients" [25]. There can be little doubt, however, that many clinicians struggle earnestly to control their emotions and judgments in order to meet these professional standards. A study of exemplar primary care physician healers found that their first commitment was "a conscious attempt... to be nonjudgmental" [26]. Indeed, one of these physicians reported, "I try to love every single patient. And I especially try to love those I initially hate." This commitment can sometimes lead to heroic professionalism, as in the case of a surgeon who dutifully treated terrorists after they had killed members of his family [27].

Although there is no social psychology literature addressing how clinicians manage their moral judgments, considerable work suggests the dynamics involved. Normative behavior can be motivated by a combination of high social consensus, *e.g.*, as evidenced in organizational and professional norms to be nonjudgmental, together with a person's acquired self-image as a moral individual [28]. Conversely, lack of social consensus or lack of strong moral identity weakens a person's motivation. Straightforward exhortations in support of professional standards have their place.

### Friend and competent, foe and incompetent, or ambivalent?

The social psychology research most obviously relevant to adverse moral judgment in healthcare is that of Susan Fiske and colleagues. Fiske has hypothesized that people stereotype groups on two different dimensions, the first being friend/foe (warmth) and the second being capability (competence) [29]. While people stereotype their own group as both warm and competent, they judge most outgroups ambivalently by ascribing both negative and positive characteristics to them. For example, Americans tend to think of rich people and feminists as cold and competent, elderly people and housewives as warm and incompetent. Very few groups*, e.g.*, homeless people and drug addicts, are stereotyped as both cold and incompetent, thus triggering contempt and disgust.

Functional neuroimaging (functional magnetic resonance imaging, or fMRI) substantiates these patterns. Under time pressure and cognitive load, white Americans seeing black faces activated their stereotypes, as measured by a priming response time test, and their amygdala, as measured on fMRI [30]. This automatic pattern evaporated, however, with a simple prompt to individuate the photos either by asking subjects to find a dot on the face or to guess whether the person liked a particular vegetable. Automatic, stereotypical responses appear to be mediated by negative emotions such as fear, anger, disgust, and contempt. Photos of homeless people and drug addicts, mixed in with other photos of people and objects, triggered disgust and activated the amygdala and insula, thought to be associated with fear and disgust, respectively [31]. Also, photos of homeless people, drug addicts, and inanimate objects failed to activate the medial prefrontal cortex, unlike all the other photos of people, thus offering a neuroscience model of dehumanization. This line of work presents powerful evidence for the impact of primitive emotional responses on social judgment, but it also begins to describe the mechanisms by which such responses are activated or not. Oldmeadow and Fiske have further found that a person's ideological beliefs moderate their stereotypes. Individuals who are motivated and informed are more likely to make nuanced judgments [32].

There is now a large social psychology and neuroscience literature describing the particular role of disgust in moral judgment [33,34]. Individuals differ in disgust sensitivity, which predicts disapproval of gay men, for instance [35]. Indeed, conservatives have higher disgust sensitivity than liberals [36]. Unsurprisingly, the cleanliness of the immediate environment influences the experience of disgust and the severity of moral judgments [37], a finding with obvious relevance to less-than-pristine safety-net healthcare settings. Use of ritual can also moderate disgust. One ethnographer found that operating room rituals do far more than merely ensure sterility. She observed surgeons to be dispassionate during surgery that exposed internal organs, secretions, pus, and feces, yet the same surgeons expressed discomfort and disgust when sitting in a darkened room watching movies of similar procedures, deprived of their protective organizing rituals [38].

### Poor people risk moral judgment

Poor patients belong to outgroups of particular interest in healthcare. Public hospitals serving these groups comprise only 2% of acute care hospitals in the United States but train 21% of doctors and 36% of allied health professionals [39]. Primary care physicians serving poor communities are often troubled by what they perceive as their patients' inadequate motivation and dysfunctional behavioral characteristics [40]. For two centuries American hospitals placated funders, physicians, and paying patients by carefully distinguishing between the deserving and undeserving poor [41]. The social medicine educators who implemented the first "patient-centered" comprehensive medical education program in 1953 stumbled against the sociological reality--not merely the self-serving stereotype--of this divide [42]. Students in this University of Colorado program were happy to care for the family-oriented working poor, who were disproportionately Black or Latino, but they often became frustrated and angry with "the chronically unemployed, the second--and in rare instances the third--generation of welfare recipients, the socially inadequate, the fatherless families, the heavy drinkers from Larimer Street, the chronic social agency clients, the unstable personalities." One student observed in exasperation, "Mr. K's recreation consists of watching the family television set with an occasional interlude of general mistreatment of wife and family."

A recent review highlighted the paucity of more current data available on medical student attitudes toward the poor [43]. As discussed below, attitudes are multifactorial complexes. In a study of 12 urban hospitals, the factors predicting whether pediatric residents felt more anger and less empathy toward underserved families were whether their clinics were well-run and whether they themselves felt effective [44]. Black residents felt more positively about both their clinics and their effectiveness. An Israeli study has looked closely at the role of physicians' international ethnicities, attitudes, and behaviors in caring for poor patients [45], but the moral complexities of interactions between international medical graduates and the diverse poor populations in the United States have received scant attention [46]. Unsurprisingly, patients at risk of moral judgment, including those who are poor, undereducated, and/or African-American, pay significantly more attention to impression management than do other patients [47].

### Medical conditions or moral issues?

As suggested by the difficult patient literature, conditions that do not readily fit the clinician's model of care and practice can place patients in moral jeopardy. Carl May and colleagues found that physicians quickly make evaluative judgments of patients' motives, the legitimacy of their symptoms, and the congruence between the physician's and the patient's conceptual model of illness [48]. Patients with chronic low back pain or medically unexplained illness, unlike those with menorrhagia, triggered negotiations that often led to unresolved contests of wills between physician and patient. Patients with problems and anxieties that could not be referred out or satisfactorily contained could trigger physician frustration and hostility. Similarly, a vignette study showed that surgeons were quick to see patients as psychologically culpable for failed back surgery, a tendency moderated by their varying levels of empathy [49].

In a study of the derogatory humor used by medical students, Delese Wear and colleagues found that obese patients were the most common targets, particularly in surgical settings [50]. In part this derision had a practical base: "Obesity makes an easy 20 minute surgery a difficult 80 minute surgery." But it also derived from students' appraisals of obese patients as blameworthy. Using the exquisite, fine-grain methodology of conversation analysis, Helena Webb has revealed the pervasive nature of moral judgment in an obesity clinic [51]. What was remarkable, moreover, was how the patients themselves described their weight loss progress in moral terms of good and bad and how actively they shaped the conversation to reflect their agency or their lack of blame. Even during the brief and seemingly simple act of weighing for primary care visits, as studied in another conversation analysis, the videotaped nurse-patient interactions revealed a discourse in which patients hold themselves morally accountable [52].

Blameworthy appraisals are more likely for conditions that appear controllable and that appear to be social as much as medical in origin. Self-injurious behavior, *e.g*., suicide attempts and cutting, tends to generate anger and frustration along with diminished optimism and effort [53], unless, notably, the clinician self-identifies as having expertise in assisting these patients [54]. Suicidal patients can also trigger frank malice and aversion, with sometimes lethal results. Inexperienced clinicians with aspirations to "heal all, know all and love all" are particularly susceptible to these antitherapeutic impulses [55]. As for patients who choose to remain in abusive relationships, a majority of clinicians may be empathetic if the patient is poor or disabled, but fewer are empathetic if the patient is an educated professional [56]. Such discrepant responses are consistent with the Fiske ambivalent stereotypes model described above, which predicts either warmth or perceived competence, but not both.

### Evidence for attitudes as complex systems

The Institute of Medicine committees on medical education and on healthcare disparities both highlighted the impact of physicians' attitudes on their patient relationships [57,58]. Evidence from diverse disciplines, however, makes it clear that a person's attitudes are neither unitary nor stable. While these studies do not tease out moral judgment from other cognitive and emotional processes, they illustrate the spectrum of methodologies that may prove useful.

Articles on countertransference in clinical medicine are often insightful but famously lack systematic empirical data. In one exception, Schwartz and colleagues showed trigger tapes of antisocial personality disorder and schizophrenia followed by use of a validated instrument that captures emotional and covert interpersonal responses [59]. Unsurprisingly, in this sample of 73 graduate level mental health professionals, the schizophrenic patient triggered warmth reactions and the antisocial patient triggered challenge and dread. In her qualitative study of a British hospital, Isabel Menzies Lyth observed, "The work situation arouses very strong and mixed feelings in the nurse: pity, compassion and love; guilt and anxiety; hatred and resentment of the patients who arouse these strong feelings; envy of the care given to the patient" [60]. Regan and colleagues studied unconscious defense mechanisms in nurses and found that immature defenses, including passive aggression, correlated with emotional exhaustion (burnout), but they themselves had significant concerns about the instrument they used [61]. Smith and colleagues have developed a method of teaching personal awareness, specifically of previously unrecognized feelings and behaviors, by having expert medical educators question and probe trainees following observed clinical interactions. Smith has been able to surface multiple fears, anxieties, and unhelpful behaviors, including unrecognized anger, disdain, and withdrawal [62].

The Implicit Attitude Test (IAT), which has made its way from social psychology into healthcare settings, depends upon subjects' response time to distinguish positive from negative unconscious attitudes. Negative explicit attitudes regarding addiction, including moral blame, are well-documented among nurses and other healthcare professionals [63,64]. In studies of physicians and nurses treating hepatitis C in injection drug users, Brener and colleagues used a version of the IAT and found that greater contact with hepatitis C patients was associated with more positive explicit attitudes but less positive implicit (unconscious) attitudes [65]. Furthermore, implicit attitudes predicted intention to change jobs (burnout) independently from the effects of explicit attitudes and job satisfaction [66].

Multiple concerns have surfaced about the IAT and related priming techniques, however, suggesting that the relationship between explicit and implicit attitudes is not yet clear [67-69]. A vignette study using the IAT found that physicians had no explicit bias against black patients but did have implicit biases that predicted their decisions to use thrombolytic treatment in the scenario [70]. A study of actual cardiac patients found that surgical recommendations varied by race for Black men, but not Black women or Hispanics, and that the effects of race were mediated by physician perceptions of patients' education and activity levels [71]. The social and psychological dynamics producing racial disparities are as yet poorly understood [72].

Neuroscience studies have shown that automatic, unconscious attitudes are highly sensitive to new information and new contexts [73]. While the amygdala produces a rapid, stereotypical initial response to a stimulus, other brain regions including the prefrontal cortex can follow with iterative reprocessing at a rate of eight cycles per second, leading to more complex and nuanced responses [74]. People who are chronically committed to egalitarian goals can inhibit stereotype activation preconsciously, that is, without awareness or new effort [75]. Social and moral intuitions shaped by extensive experience and learning can operate without any conscious awareness [76].

Researchers no longer think of moral judgments as exclusively cognitive [77]. Even those who minimize the emotional component acknowledge that emotions play a powerful role in motivating relevant action [78]. Using functional MRI, we can now visualize the neural substantiation of moral emotions. Guilt and compassion activate different distributed brain networks than do disgust and indignation [79].

The simple stimuli and vignette studies that are stock-in-trade for social psychologists and neuroscientists are appropriate for research on first impressions in healthcare, where encounters with strangers increasingly characterize the patient experience [80]. But negative appraisals may develop dynamically over the course of an encounter and may arise, change, or disappear as encounters become relationships, much as Goffman described for the discovery and management of stigma [81]. Fortunately, social psychologists themselves recognize the need for more naturalistic research strategies [82,83]. Subjects in an immersion virtual environment may respond as if the situation is real, as shown by a virtual replication of the Milgram obedience experiment [84]. Neuroscientists have begun to use videogames as stimuli for subjects in functional MRI scanners [85]. Within healthcare, Debra Roter and colleagues have called for more creative simulations to investigate emotion and non-verbal behavior, not only by using standardized patients but also by having patients or clinicians respond to vignettes and videotaped interactions [86].

### Variation among clinicians

Studies of clinician personalities, habits, and skills have offered only modest insights into variations in moral judgment. One study of medical student Machiavellianism--itself predicted by male gender, authoritarianism, intolerance of ambiguity, and external locus of control--found that Machiavellian students had more negative attitudes toward geriatric and hypochondriac patients [87]. Benjamin Chapman and colleagues studied community primary care physicians in Rochester, Minnesota, using personality tests and audiotaped standardized patient visits, and found that personality characteristics shaped their interviews of depressed patients [88]. Personality explained only 4-7% of the variance, however, while physician demographic factors explained only another 4-7%. The authors note that "patients who are belligerent, distressed, passively noncompliant, medically complex, or particularly knowledgeable, congenial, and appreciative" might thereby influence communication, but their study did not examine that influence.

Emotional intelligence measures appear to be emerging from an infancy troubled by methodological concerns and popular hype [89], but their application in healthcare [90-92] has yet to focus on difficult patient relationships. One pilot study of psychiatric patients and staff suggested that attachment theory may predict the success of therapeutic relationships: lower attachment anxiety and avoidance scores of staff members correlated with better relationships as rated by patients [93]. Medical students with secure attachment styles are more likely to seek relationship-focused primary care residencies [94].

Gender is often a factor in clinical relationships, as illustrated by Hall's reciprocal likability study cited above in which female physicians said they liked their patients more than male physicians did. Patients agreed that female physicians liked them better and also said that they liked female physicians more than they liked male physicians [13]. We do not know, however, how gender may figure in clinicians' moral judgments of patients. Sonia Crandall found that female medical students had more favorable attitudes toward poor patients than male medical students [95], although later studies have not replicated this finding [44,96]. Women, including female physicians, generally score higher than men in empathy, but we still lack evidence that such differences are of consequence in clinical interactions [97]. In a laboratory study using emotional videos as triggers, women had greater responses than men in empathy, monetary generosity, and oxytocin levels [98]. In addition to its roles in birth, lactation, and mother-infant bonding, oxytocin has complex effects upon the brain and social behavior in females and males [99], but its roles in moral emotions are far from clear [100,101]. A single intranasal dose of oxytocin in male students increased their trust and social risk-taking in interactive dyads [102]. Carole Gilligan's hypothesis that women and men employ different modes of moral reasoning (care versus justice) has failed to garner strong support [103]. Indeed, Kohlberg's theory of developmental moral stages, on which Gilligan's work depended, has itself been significantly modified [104] or abandoned [77]. Nevertheless, studies continue to appear with evidence of gender differences in emotionally-charged moral appraisals [105]. Using functional neuroimaging, Carla Harenski found that even when women and men make similar moral judgments, they may do so via different dynamics, activating different brain circuitry [106].

Diverse studies cited here demonstrate the folly of expecting unifactorial attributes of either patient or clinician to dominate moral judgments. Once the clinician is engaged in dyad and context, a complex system is in play. In the course of this review I asked colleagues how they managed their own moral judgments of patients. Two simple examples illustrate these dynamic, often conflicted appraisals. An experienced midwife working in public sector explained that she felt little moral judgment while caring for wayward pregnant women and girls. "I'm okay with my patients," she said, "but I have trouble with some of their partners when they appear." A family practitioner also reported that she had no difficulty with the women who return multiple times with the consequences of high-risk behavior. But upon learning that a patient had a prison background, "I had to ask why. It was alright with me if he lied about it, but I had to ask." One might ask why truth-telling itself rises so infrequently as a moral issue in the minds of clinicians. In response to continual invasive questioning, patients routinely withhold, distort, and otherwise injure the truth, either consciously or not, and yet within the clinical relationship and its contextual frame, such violations usually do not merit moral notice and do not trigger moral appraisals.

### The power of setting and organizational factors

Organizational factors may weigh more than individual skills and attitudes in the salience of moral judgments, as suggested in Bowers' study of facilities for criminal patients with personality disorders [20]. In the University of Colorado teaching program described above, frustration with the poor and behaviorally difficult patients occasionally escalated into hostility from students and teaching staff [42]. The researchers noted that such hostility was "almost always temporary," however. "The over-all attitude of the clinic staff toward patients created an atmosphere in which hostile behavior was immediately conspicuous."

The importance of setting was obvious in my informal survey of colleagues. Those in correctional healthcare had learned how to compartmentalize and/or minimize their appraisals. After all, a cooperative and appreciative patient with Nazi tattoos is still a cooperative, appreciative patient. For those in middle-class or well-to-do clinical settings, on the other hand, the issue of morally reprehensible patients rarely arises. I did not find evidence in the literature to support Jodi Halpern's assertion that, once physicians have invested in caring for patients, they then "invest a great deal in idealizing" them so as to avoid their own negative emotional reactions [107]. Halpern may be right nevertheless. Clinicians with middle-class practices generally do not ponder what percentage of those men and women have molested children, whereas moral issues are quite salient where convicted criminals come labeled as such. Idealizing distorts clinicians' perceptions and thus limits biopsychosocial comprehension, but it may help motivate clinicians to provide good care. In some cases, it may also protect patients from clinicians who would be unprepared for their own reactions to a more complete picture.

The landscape between prison and polite suburbia, based on the literature reviewed above and confirmed by my own interviews, is one of great variation. I certainly heard people assert that moral appraisals were off-limits and best avoided. "You can't go there." Most of my respondents acknowledged that when a strong moral judgment arises, it comes with palpable emotional impact, sometimes accompanied by a change in strategy. "I go numb, I just shut down." "I put up walls." "I switch gears." They described shifting from emotional engagement to duty, "falling back on professionalism as a floor." Frequently I heard some version of, "It depends." Moral judgments of varying intensity are activated or not, and are problematic or not, depending upon a complex interplay among clinicians, patients, tasks, and organizational factors. We know little about when such judgments occur, what impact they have, which corticolimbic "gears" get "switched," how, and why.

### Promising subjects, settings, and strategies

Progress in understanding professional development and skills often emerges from close studies of experts [108]. Recent empirical studies have focused on clinicians chosen for expertise in de-escalating aggressive psychiatric patients [109], in role-modeling humanistic bedside behavior [110], and in discussing advance directives [111]. Expertise may be largely tacit [112] and embodied in habits that operate automatically without conscious intention [113]. Such expertise may be more or less reliable [114,115]. William Branch and colleagues, noting that well-intentioned traditional ways of teaching respect to students generally fail, have begun gathering empirical data on expert approaches to modeling and teaching humanistic behavior [110,116,117]. They have not, however, addressed issues of moral judgment, nor have they focused on challenging safety-net and other stigmatized settings.

Even without knowing exactly what *should *happen, understanding what *actually *happens in such settings would be invaluable. Safety-net settings concentrate extreme outgroups, *e.g.*, homeless alcoholics and drug addicts, as well as groups that trigger ambivalent stereotypes, thus offering challenges that often disconcert or ensnare clinicians [118]. Who decides to work in such settings, who performs well there, and who survives over the long haul? With experience and age, many people improve their emotional regulation and more skillfully defuse negative situations [119]. Do clinicians demonstrate these improvements over the course of their careers?

Most of us can point to role models who move with such capacious modesty, competence, and wisdom that patients (and trainees) respond with their better humanity. These role models seem to take in, contain, and transmute negativity and pain. It would be helpful to know the resources they bring to bear and the strategies they use.

Research on emotion regulation may help us understand how clinicians manage moral judgment. We can regulate our emotions by focusing attention to task, as implied by the dirty work studies cited above, or by reappraising the situation. Cognitive reappraisal appears to have healthier personal and interpersonal consequences than emotion suppression strategies [120]. A simple suggestion to control one's feelings can decrease disgust reactions to a stimulus. The same stimulus will increase autonomic stress markers when the instruction is to hide (suppress) one's feelings [121]. Emotion regulation can also occur via more global strategies [122]. Mindfulness training, which has well-established physiological and psychological benefits [123], can lead to improvements in physicians' wellbeing and strengthening of their patient-centered attitudes [124]. Evidence for the multilevel benefits of narrative expression, as described for Balint groups [125] and narrative medicine [126], is also beginning to emerge from controlled studies in patient populations. Several brief exercises in expressive writing have increased CD4+ counts for HIV patients [127] and have decreased infirmary visits for incarcerated sex offenders [128]. Creation of narratives facilitates integration of experience into cognitive frameworks, thereby down-regulating disturbing emotions. Paramedics learn to cope with gore and danger using cognitive strategies and organizational and interpersonal support, but they report long-lingering distress from events that they could not "make sense of" and integrate into coherent stories [129].

### Is interest as an essential quality?

One of the factors that may prevent clinicians from triggering moral appraisals is interest, often equated with curiosity. Recall that a subject in the Fiske experiment could avoid lighting up her amygdala by focusing on whether the person in the photo liked a certain vegetable or by looking for a dot on his face [30]. Good teachers have stressed the value of curiosity for clinical care [130,131]; one of my teachers insisted that "every cirrhotic is different." Writers that quote from Peabody's lecture, as I did in the opening paragraph, often neglect the independent clause of his final sentence: "One of the essential qualities of the clinician is interest in humanity, for the secret of the care of the patient is in caring for the patient." With current social psychology methodologies, Peabody's assertion about the critical role of interest is now testable.

Several sociologists have long included interest, surprise, and boredom in their work on emotions [132]. Social psychologist Paul Silvia has recently posited that interest depends upon a combination of the stimulus complexity and a person's appraisal of her ability to comprehend and cope with the stimulus [133]. Once a stimulus--or perhaps patient, for our purposes--appears beyond one's comprehension and ability to manage, interest wanes. These appraisals mediate individual personality differences in curiosity and the experience of interest [134,135]. Carol Sansone points out that we can use interest to self-regulate our motivation. When intrinsic motivation lags, we can activate strategies to engage our interest and thereby remain motivated for the task [136]. When all else fails, we can try to take interest in our own boredom, a classic maneuver in reflective practice. One of the respondents in my informal survey, a psychologist with a police background, described his strategy in leading therapy groups for sex offenders, some of whose victims he had met and interviewed: "My goal is to love them [the inmates], and if I can't do that, I at least try to love the process."

### Finding one's way in a looking glass world

The pattern that emerges from this composite literature suggests a looking glass world nested within a looking glass world, each paneled with both true and distorting mirrors. In the broader moral communities outside of healthcare, we make legitimate moral judgments of each other's behavior on a continuum from virtuous to contemptuous. Sustained community contributions garner our praise; child abuse, our censure. We also make distorted-mirror judgments that are demonstrably inaccurate and illegitimate because of stereotypes derived from a host of factors such as age, gender, ethnicity, wealth, and power. The legitimate and illegitimate appraisals often reflect and interact, begetting uncertainty.

Within the healthcare setting, barring incapacity, patients remain moral agents and retain accountability, so their behavior in this setting is also subject to legitimate moral judgments. Patients' violent or racist behavior within healthcare facilities, for instance, arouses clinicians' legitimate disapproval and triggers sanction. The illegitimate, distorted-mirror judgments specific to healthcare include noncompliance with unrealistic or inappropriate instructions. Clinicians frustrated by such non-compliance may label patients "bad."

For better and worse, clinicians are human, so moral and social judgments from the broader community can penetrate into the world of healthcare relationships. The good news for patients who may appear morally dubious is that clinicians who feel effective are often happy to overlook transgressions irrelevant to the healthcare task. Within the bounded and ritualized healthcare setting, these "outside" judgments may be inactivated or immaterial, especially if the patient presents with a readily remediable problem, *e.g*., a minor or straightforward surgical procedure. In spite of clinicians' egalitarian beliefs and professionalism, however, it is unrealistic to expect that all their moral and social appraisals of patients as human beings will be immaterial. As noted by Glaser and Strauss, favored patients are more likely to get "more than routine care" and less-favored patients, "less than routine care" [9].

Moving from one study to another in these looking-glass spaces has a yes/but quality reflecting these legitimate and illegitimate judgments, a quality sometimes evident within a single study. Joan Cassell, who in the introduction described ICU nurses making judgments of patients, observed that the surgeons and intensivists attempted to be unaware of patients' stories and to reduce their work to technical, biomedical tasks [3]. Isabel Menzies Lyth described similar strategies among nurses [60]. "The enterprise proposes to toss out the baby, the moral dimension of medicine, with the bathwater, moral judgments of patients," noted Cassell. "I believe that even in the highly charged, technologically driven atmosphere of the SICU, the effort to perceive the patients as persons, to attend to their stories rather than ignoring them, leads to more human and humane care--of patients and their families." We have no evidence to support her belief, however, whereas she and others offer ample evidence that more personalized knowledge of patients can place them in jeopardy of clinicians' moral judgments.

### Why have we neglected research on moral judgment?

While speculative, explanations of the research neglect of moral judgment in healthcare could facilitate understanding and progress in this domain. Researchers may have assumed that bioethicists own the topic or that it requires literacy in moral philosophy. The isolation of the medical and nursing literatures from mainstream sociology and psychology may play a role. It is also true that researchers and clinicians who concentrate on criminals or other deviant populations in jeopardy of moral judgment are themselves in jeopardy of what Goffman called "courtesy stigma" [81]. Thus legitimate concern about risk to reputation might dissuade some from exploring this terrain. Finally, given that healthcare leaders colluded in America's long history of dividing patients into deserving and undeserving groups, most modern educators and professionals have emphasized a nonjudgmental egalitarianism in which clinicians' moral appraisals are taboo. Taboo or not, they are pervasive, and patients know this. Hall's likability study demonstrates that patients are exquisitely sensitive even to unspoken and perhaps unconscious appraisals. Egalitarian professionalism puts a brave face over a complex reality. Nursing in particular is beset by a caring ideology that discourages frank examination of what nurses actually feel and do [137].

The issue of poverty illustrates the legitimate *versus *illegitimate looking-glass complexity of moral judgment. As noted in the review cited above, we have remarkably limited data on clinician attitudes to poor people [43]. Even in the data we have, there is a gap with regard to moral judgment. In their review, Wear and Kuczewski note that a majority of Americans give more credence to individual, blameworthy causes of poverty than to social or structural causes; they also note that such individualistic attributions are more common among right-wing or conservative Americans. The thrust of their article is toward overcoming stereotypical, illegitimate biases against the poor, but they never acknowledge the possibility that some people's behaviors may indeed be responsible for their poverty. Researchers in the 1950 s University of Colorado study discussed earlier described how faculty and students were frustrated by the individual behavior patterns of some poor individuals and families. Such assessments and frustrations seem to have dropped off the healthcare research agenda. The only article I could find from the past two decades directly addressing physician explanations of poverty was from Belgium [40]. The physicians serving a high-poverty area there showed commendable empathy, understanding, and concern for their patients, and they cited sociopolitical explanations of the poverty. They cited psychological and individual explanations as well, however, most commonly patients' lack of ambition and motivation to improve their situation but also addiction, laziness, and lack of skills or intellectual capacity.

There is reason to be cautious in this looking-glass space, subject as it is to subtle and not-so-subtle distortions. Many poor people, while themselves suffering from inaccurate, cruel, and damaging stereotypes, attribute the poverty of other poor people to individual behavioral causes [138]. More seriously, theories of the culture of poverty and the underclass, originally advanced by liberal sociologists, were used by journalists and politicians to blame and demonize the poor [139]. Sociology is only now recovering enough to get beyond an individual *versus *structural either/or dichotomy toward an understanding of the diversity within poor communities, even within poor families, and the interplay of malignant structural forces with bad behavior at the individual level [140].

A final reason for caution and discernment is the overlap of morality and convention. In naturalistic settings, unlike the laboratory, the line between moral and non-moral violations of expectations and trust is often not sharp. Philosopher Margaret Urban Walker reminds us that our everyday lives are littered with small, ordinary violations to which we take offense, some of which are moral, others more conventional [141]. From the sociologists we know that clinicians' moral and social appraisals often mingle together, and from the neuroscientists we know that moral reactions, such as disgust, may ride non-moral, phylogenetically ancient pathways. Healthcare's avoidance of this complexity has not improved our understanding of it.

## Conclusion

While incomplete, the research base leaves no doubt that clinicians make moral judgments of their patients. We know almost nothing about the prevalence of negative judgments or about their impact on patient care and on the clinicians themselves. We do not know which combinations of patient, clinician, and situational factors trigger negative moral appraisals. We have yet to wonder whether those appraisals follow a linear or curvilinear line from negligible to substantial impact. Well-meaning advocacy of nonjudgmental attitudes and patient-centered care is not likely to achieve its goals if it discourages understanding what actually happens in healthcare relationships. On the positive side, we have a wealth of data from social psychology and neuroscience that is beginning to demonstrate how moral judgment emerges from the interplay of rival brain neurocircuits in the context of human interactions. Research methodologies from these sciences are available to facilitate the study of moral judgments in healthcare. We are now in a position to explore how healthcare relationships arise through our biologically makeshift brains and bodies and how organizational cultures modulate those relationships. Improvements in the empirical research base will inform clinicians' efforts to minimize stereotyping and stigma, honor their own moral feelings and beliefs, and stay engaged with the patients they find morally troubling.

## Competing interests

The author declares that he has no competing interests.

## Authors' contributions

The author is solely responsible for this manuscript.

## Author's information

Terry Hill, MD, FACP, is an Assistant Clinical Professor in the Department of Medicine at the University of California, San Francisco. A geriatrician, he has spent most of his career with stigmatized patients in stigmatized settings, *e.g*., nursing homes and safety-net urban hospitals. His most recent position was Chief Executive Officer for Medical Services, California Prison Healthcare Receivership.
